# Biochemical and Biotechnological Approaches to Disease Diagnosis and Therapeutics: A Comprehensive Review

**DOI:** 10.7759/cureus.107864

**Published:** 2026-04-28

**Authors:** Pallavi Kadwe, Lipika Jena, Diptimayee Jena, Yugantar Wakde, Sadhana Kakaso Pawar, Deeptak Biswas

**Affiliations:** 1 Department of Biochemistry, N.K.P. Salve Institute of Medical Sciences and Research Center, Lata Mangeshkar Hospital, Nagpur, IND; 2 Department of Microbiology, Institute of Medical Sciences and Sum Hospital, Bhubaneswar, IND; 3 Department of Biochemistry, Maharaja Krushna Chandra Gajapati Medical College and Hospital, Berhampur, Berhampur, IND; 4 Department of Biochemistry, Shri Rawatpura Sarkar Institute of Medical Sciences and Research, Naya Raipur, IND; 5 Department of Biochemistry, Department of Biochemistry, Bharati Vidyapeeth Deemed to be University Dental College and Hospital, Pune, IND; 6 Department of Microbiology, Kalyani Mahavidyalaya, University of Kalyani, Nadia, IND

**Keywords:** bioinformatics, biotechnology, diagnostics, precision medicine, therapeutics

## Abstract

Advances in biochemical and biotechnological sciences have transformed modern medical practice, enabling earlier disease detection and more precise therapeutic interventions. The increasing complexity of oncological, infectious, metabolic, and degenerative diseases necessitates diagnostic and therapeutic systems capable of accurately interpreting subtle molecular variations. This review synthesises key innovations in biochemical diagnostics and biotechnology-driven therapeutics, highlighting their clinical relevance and contribution to precision medicine. Recent advancements have significantly improved early disease identification, molecular characterisation, and targeted treatment strategies. These approaches enable minimally invasive monitoring, enhance diagnostic accuracy, and support personalised clinical decision-making across diverse disease conditions. Overall, biochemical and biotechnological innovations are central to advancing precision medicine, and continued validation, optimisation, and integration into healthcare systems will be essential for improving clinical outcomes and sustaining long-term progress.

## Introduction and background

The complexity of biological processes that regulate the development, course, and maintenance of complex illnesses such as malignancies, chronic metabolic disorders, infectious diseases, and neurodegenerative syndromes has been increasingly recognised and further elucidated by advances in medical science [1]. These conditions arise from complex interactions among genetic, biochemical, immunological, and environmental factors. Conventional diagnostic models based on clinical manifestations or morphological features fail to identify subtle molecular imbalances that precede the onset of symptoms [2]. These limitations result in delays in risk assessment, inaccurate stratification, and reduced opportunities for timely therapeutic intervention [3]. Growing insights into molecular pathophysiology have shifted clinical focus toward diagnostic strategies based on biochemical analysis and biotechnology-driven platforms [4]. Techniques based on genomics, transcriptomics, proteomics, and metabolomics provide a comprehensive understanding of disease-related molecular signatures. These approaches, collectively referred to as multi-omics technologies, enable the early detection of abnormal metabolic responses, protein misfolding, nucleic acid variants, and immune dysregulation patterns [5]. Consequently, biochemical diagnostics is becoming increasingly dominant in conditions that involve high-resolution molecular profiling, especially in diseases characterised by significant biological heterogeneity [6].

Simultaneous advances in biotechnology have expanded the range of therapeutic approaches capable of targeting disease processes at their molecular origins [7]. Clustered Regularly Interspaced Short Palindromic Repeats (CRISPR)-based genomic modulation, monoclonal antibody therapies, engineered immune platforms, nanoparticle-based drug delivery systems, recombinant protein therapeutics, and synthetic biology constructs are examples of emerging technologies that demonstrate significant potential for precise intervention [2]. These modalities are designed to enhance target specificity, reduce systemic toxicity, and overcome therapeutic resistance in complex diseases [1]. Despite these advancements, significant gaps still exist that limit the widespread implementation of biochemical and biotechnological innovations in routine clinical practice [8]. Most molecular assays require specialised equipment, controlled laboratory environments, and advanced computational support, which restrict their use in resource-limited settings [6]. Diagnostic biomarkers often exhibit variability across populations, making it difficult to establish standardised thresholds or universal clinical cut-off values [9]. Additionally, disparities in infrastructure, cost constraints, and variations in workforce expertise contribute to unequal adoption across healthcare systems.

Similar limitations affect the application of biotechnology-based therapeutics. Therapies involving gene modulation, nanomaterial carriers, or engineered cells raise concerns regarding long-term safety, immunogenicity, off-target effects, and scalability of production [4]. Regulatory frameworks are continuously evolving with the development of new technologies, but a uniform assessment standard remains an urgent requirement. Longitudinal datasets for tracking therapeutic performance, biological compatibility, and delayed adverse events remain limited, resulting in uncertainty regarding long-term outcomes across different populations [8]. The emergence of precision medicine has highlighted the importance of integrating biochemical diagnostics with advanced biotechnological therapies. Multi-omics convergence, predictive bioinformatics models, microfluidic diagnostic devices, and machine learning- and deep learning-driven analytics represent a shift toward personalised clinical care [10]. These systems are designed to predict disease susceptibility, treatment response, and optimal therapeutic pathways with greater accuracy than traditional approaches [3]. Precision medicine has the potential to transform clinical practice by enhancing diagnostic certainty and treatment efficacy through the integration of robust molecular evaluation and targeted intervention strategies.

The growing clinical need for minimally invasive diagnostics, targeted therapeutic delivery, and patient-specific treatment approaches underscores the importance of integrating knowledge from molecular biology, analytical chemistry, biomedical engineering, and clinical medicine [10]. A clear understanding of biochemical pathways involved in disease pathogenesis and progression is essential for developing biotechnology-based therapeutic strategies with improved specificity and reduced systemic effects. Comprehensive insight into these interrelated systems supports the development of diagnostic frameworks that enhance early detection and enable treatment strategies tailored to individual biological variability [11]. A critical evaluation of these biochemical and biotechnological approaches is necessary to identify both their strengths and existing translational limitations. This overview can contribute to improving diagnostic accuracy, facilitating the development of more targeted therapies, and promoting the adoption of evidence-based technological innovations across diverse healthcare settings. The continued evolution of these technologies is likely to play a critical role in shaping future clinical practice, particularly in high-burden diseases that require early detection, molecular risk assessment, and individualised therapeutic interventions. This review adopts a comprehensive, cross-disease framework, addressing a broad spectrum of chronic and acute diseases across multiple clinical domains to evaluate their clinical relevance and translational potential.

Objectives of the review

This review aims to present an integrated analysis of biochemical modalities that support advanced disease detection and monitoring, along with biotechnological strategies designed to achieve targeted therapeutic action. This synthesis encompasses a broad, cross-disease perspective, incorporating multiple categories of chronic and acute diseases rather than focusing on a single disease entity. The objective is to clarify the scientific principles, clinical relevance, translational limitations, and prospective developments associated with these approaches to enhance understanding of their impact on contemporary medical practice.

Methodology

Study Design

The review strategy was designed to provide a comprehensive synthesis of biochemical and biotechnological advancements in disease detection and treatment. This work follows a structured narrative review approach aimed at integrating diverse and clinically relevant evidence rather than a protocol-driven systematic framework.

Data Sources and Search Strategy

Relevant literature was identified through searches of major scientific databases, including PubMed, Scopus, Web of Science, and Google Scholar, using appropriate biochemical and biotechnological keywords. Search terms included combinations of “biochemical diagnostics,” “biotechnology in therapeutics,” “molecular diagnostics,” “multi-omics,” “gene therapy,” “nanobiotechnology,” “biosensors,” and “precision medicine.” Additional sources were incorporated through manual screening of reference lists to ensure broader coverage of pertinent studies.

Eligibility Criteria

Emphasis was placed on peer-reviewed English-language publications with clear clinical or translational relevance. Studies were selected based on their scientific quality, relevance to the topic, and contribution to advancing understanding of diagnostic and therapeutic innovations. Priority was given to original research articles, clinical studies, systematic reviews, meta-analyses, and translational investigations with validated or clinically interpretable findings. Editorials, conference abstracts, non-peer-reviewed content, and studies lacking clinical applicability were excluded.

Study Selection Process

The selection process involved an initial identification stage followed by relevance-based screening of titles, abstracts, and full texts. Studies were further categorised thematically to ensure inclusion of scientifically robust and clinically meaningful evidence aligned with the objectives of the review.

Risk of Bias Assessment

To enhance methodological rigour, the risk of bias and study quality were evaluated by two independent reviewers. Each study was assessed based on methodological clarity, validity of results, and relevance to clinical application. Any discrepancies between reviewers were resolved through discussion and consensus to ensure consistency and minimise subjective bias in study selection.

Data Synthesis and Analysis

Data synthesis was conducted using a qualitative and integrative approach, involving comparative evaluation, thematic grouping, and critical interpretation of findings across selected studies. This method enabled the identification of key trends, technological advancements, and translational challenges within the field.

Methodological Overview

This approach enabled a structured yet flexible synthesis of current evidence, consistent with the principles of a comprehensive narrative review. Table 1 summarises the methodological workflow for literature identification, screening, and synthesis, providing a clear overview of each stage.

**Table 1:** Summary of Literature Selection and Review Process

Stage	Description
Literature identification	Database search (PubMed, Scopus, Web of Science, Google Scholar) using relevant keywords
Supplementary search	Manual screening of reference lists for additional relevant studies
Screening criteria	Selection based on relevance, scientific quality, and clinical/translational significance
Inclusion focus	Original research, clinical studies, systematic reviews, meta-analyses, translational studies
Exclusion criteria	Editorials, conference abstracts, non-peer-reviewed content, studies without clinical relevance
Synthesis approach	Thematic and integrative analysis consistent with a comprehensive narrative review

## Review

Advances in molecular diagnostics and biomarker discovery

The development of molecular diagnostics has significantly improved disease diagnosis by enabling the detection of nucleic acid alterations, gene expression changes, and epigenetic modifications that occur during early pathological progression [12]. Assays based on deoxyribonucleic acid (DNA) and ribonucleic acid (RNA) allow accurate characterisation of single-nucleotide variations, chromosomal rearrangements, microbial genomic fragments, and transcriptomic perturbations associated with clinical deterioration [5]. Next-generation sequencing technologies, polymerase chain reaction (PCR) platforms, and quantitative amplification systems provide high levels of analytical sensitivity for identifying rare molecular events across diverse disease contexts [9]. These platforms facilitate the early identification of oncogenic mutations, antimicrobial resistance genes, hereditary risk variants, and pathogenic genomic material using minimal sample volumes. Liquid biopsy is a valuable advancement in molecular diagnostics, applicable to circulating tumour DNA, extracellular vesicles, tumour-derived RNA, and circulating tumour cells [4]. This minimally invasive approach enables real-time monitoring of molecular changes associated with tumour evolution, metastatic activity, and therapeutic exposure. Circulating biomolecules reflect clonal diversity, mutation burden, and residual disease, allowing continuous monitoring without the procedural limitations of traditional tissue sampling [13].

Another important aspect of diagnostic innovation is the use of epigenetic biomarkers. Early alterations in gene regulation, including changes in DNA methylation patterns, histone modifications, and chromatin accessibility, are indicative of malignancies, inflammatory conditions, metabolic disorders, and neurodegenerative diseases. These biomarkers support early detection, prognostic evaluation, and therapeutic stratification [8]. Stable circulating nucleic acid-based epigenetic signatures enable the development of highly specific assays for early detection, prognosis, and therapy stratification, while also enhancing the interpretation of diagnostic data beyond static genetic variation [10]. The integration of proteogenomic data enhances biomarker discovery by linking genomic alterations with downstream protein expression patterns and signalling dynamics [14]. This integrated approach improves the identification of functional biomarkers that accurately reflect disease biology. Such markers can predict therapeutic response, identify resistance mechanisms, and support precision treatment strategies in oncology, infectious diseases, and immune-mediated disorders. Multiplex molecular panels further extend diagnostic capability by enabling the simultaneous analysis of interacting biomarker networks within a single analytical platform [8]. The adoption of molecular diagnostics has expanded significantly as platforms become more automated, accessible, and analytically robust [2]. Genomic, transcriptomic, and epigenomic predictive biomarkers support early intervention, improved prognostic assessment, and therapeutic planning. Molecular diagnostics is expected to continue playing a central role in future clinical practice, supported by ongoing advancements in assay chemistry, high-throughput technologies, and computational analysis.

Proteomics and metabolomics in disease characterisation

Proteomics and metabolomics complement each other in elucidating molecular changes associated with specific disease states [11]. Proteomic profiling measures variations in the abundance, structure, and post-translational modifications of proteins that influence cellular behaviour under pathological stress [9]. Early biochemical imbalances often result in alterations in phosphorylation, glycosylation, oxidation, and conformational stability, which are indicative of malignancy, metabolic dysregulation, immune dysfunction, or neurodegenerative disorders [15]. High-resolution mass spectrometry (HRMS), multiplex protein analysis, and targeted proteomic platforms provide high sensitivity for detecting disease-specific protein signatures that support accurate staging and guide therapeutic decision-making [5]. Metabolomics provides additional insight by mapping small-molecule intermediates that reflect dynamic metabolic processes. Alterations in amino acid, lipid, organic acid, and nucleotide derivative profiles are observed in tumour progression, infectious diseases, inflammatory responses, and endocrine disorders [9]. Metabolite profiling, based on nuclear magnetic resonance (NMR) spectroscopy and mass spectrometry, is highly informative as it identifies changes in oxidative stress, mitochondrial function, substrate utilisation, and enzymatic activity [7]. These biochemical signatures are valuable for distinguishing disease subtypes and detecting early functional disruptions that may not be identified through structural imaging or conventional assays [16].

Integrated analysis of proteomic and metabolomic data enhances disease characterisation by linking upstream protein regulation with downstream metabolic flux. This combined approach improves pathway mapping, facilitates biomarker discovery, and enables more precise identification of biological drivers associated with disease progression or therapeutic resistance [5]. Pattern recognition tools and multivariate analytical methods further refine interpretation by identifying coordinated molecular changes within interconnected pathways. The expanding application of proteomics and metabolomics in clinical settings reflects their growing importance in diagnostic accuracy, early detection, and therapeutic planning [17]. Recent advancements in analytical instrumentation and the harmonisation of multi-omics approaches continue to enhance the clinical utility of these platforms in modern disease assessment. Table 2 presents a comparison of the core features of proteomics and metabolomics.

**Table 2:** Key Features of Proteomics and Metabolomics MS: mass spectrometry, NMR: nuclear magnetic resonance, LC-MS: liquid chromatography-mass spectrometry, GC-MS: gas chromatography-mass spectrometry

Feature	Proteomics	Metabolomics	References
Molecular target	Proteins	Small metabolites	[16]
Analytical insight	Pathway regulation	Metabolic flux	[2]
Core tools	MS, protein arrays	NMR, LC-MS, GC-MS	[17]
Clinical utility	Staging, target identification	Early function	[12]

Micro-analytical platforms for rapid diagnosis

Biosensors, biochips, and lab-on-a-chip systems have transformed diagnostic practices by enabling faster, more sensitive, and minimally invasive detection of biomolecular targets [13]. Biosensors operate on the principle of converting biochemical interactions into measurable electrical, optical, or thermal signals, which indicate the presence or concentration of specific analytes [18]. Enzyme-based, immunological, nucleic acid-based, and cell-based biosensors provide robust detection systems for pathogens, metabolic markers, toxic substances, inflammatory mediators, and tumour-associated proteins [10]. High signal specificity is achieved through engineered recognition elements that selectively bind to target biomarkers, making these systems particularly valuable for point-of-care applications [13]. Biochips further advance diagnostic capabilities by offering high-density microarray platforms in which nucleic acids, proteins, or metabolites can be detected at microscale resolution. DNA microarrays, protein chips, and glycan arrays enable high-throughput profiling of gene expression patterns, protein-protein interactions, mutational signatures, and immune responses [6]. These systems allow the simultaneous analysis of multiple biomarkers within a compact analytical platform, making them suitable for rapid molecular stratification. Their small size also improves reagent efficiency and facilitates use in resource-limited settings or when sample volumes are restricted [19].

**Figure 1:**
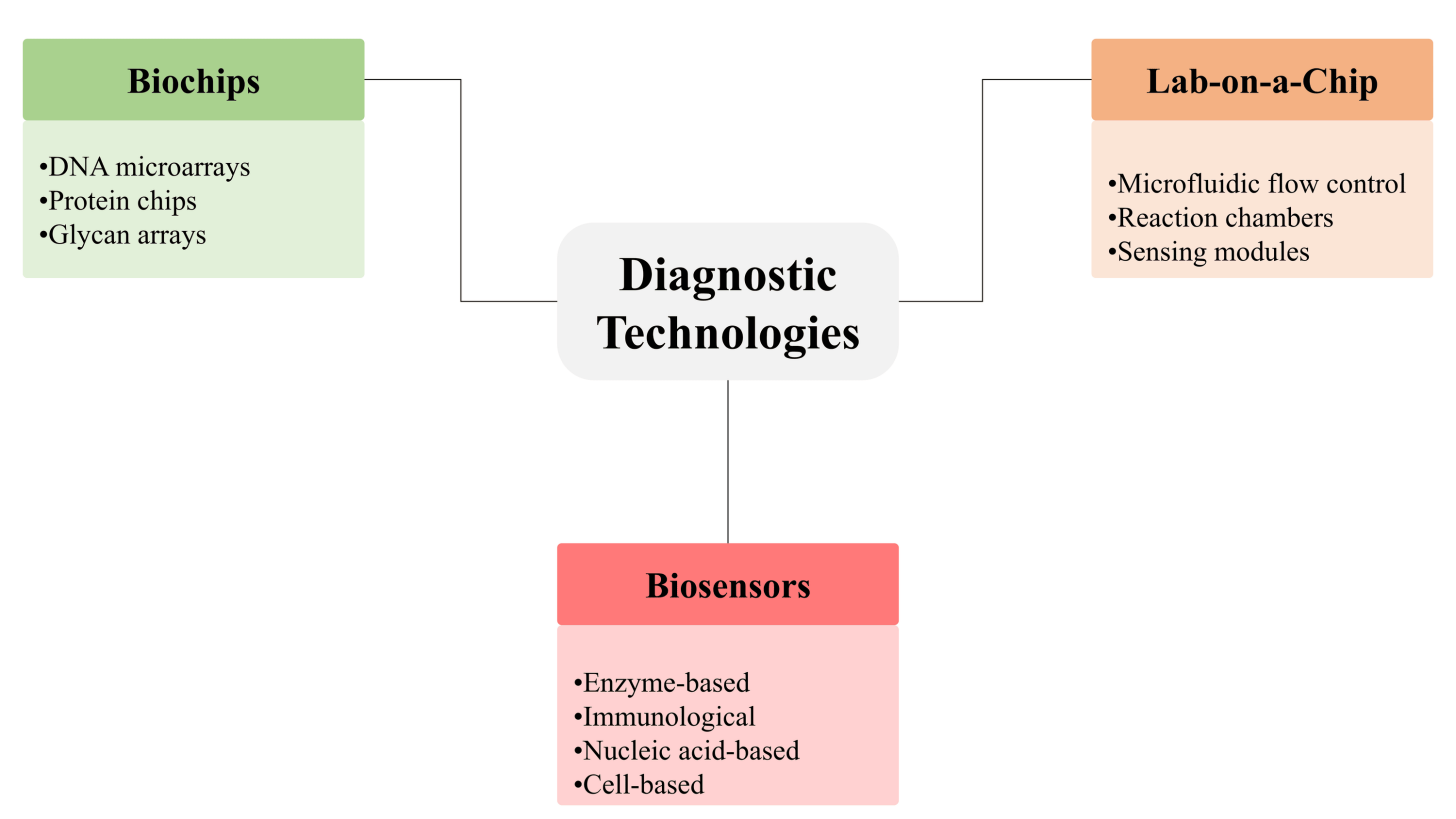
Micro-analytical Platforms in Modern Diagnostics DNA: deoxyribonucleic acid Created by authors using Microsoft PowerPoint

Lab-on-a-chip technologies integrate microfluidics, reaction chambers, and sensing components into compact devices capable of performing complex biochemical operations on a single platform [15]. Microfluidic systems enable precise control of nanolitre to microlitre sample volumes, supporting rapid reactions, cell sorting, pathogen isolation, and nucleic acid amplification [9]. These platforms enable near-real-time diagnostic outputs for infectious disease screening, metabolic profiling, and genetic analysis. Miniaturisation reduces processing time and operational complexity, enabling deployment in decentralised healthcare environments, emergency settings, and field diagnostics [13]. Integration of biosensing elements with microfluidic networks further enhances diagnostic performance by improving sensitivity, reducing contamination risk, and enabling multiplexed detection. Advances in nanomaterials, surface functionalisation, and microfabrication have improved signal stability, lowered detection limits, and enhanced device performance [16]. Continued innovation in biosensor-chip integration is expanding the scope of point-of-care diagnostics, facilitating earlier disease detection, accurate monitoring, and improved clinical decision-making across diverse medical applications [20]. Figure 1 illustrates the core diagnostic technologies, including biosensors, biochips, and lab-on-a-chip systems.

CRISPR-based diagnostic platforms and their medical applications

CRISPR-based diagnostics have introduced a paradigm shift in molecular detection through the use of programmable nuclease systems capable of recognising specific nucleic acid sequences with high precision [18]. CRISPR-Cas complexes identify target DNA or RNA through guide-specific complementarity, generating signal outputs that indicate the presence of genetic or pathogenic material [21]. This mechanism forms the basis of several diagnostic systems designed to provide rapid, sensitive, and low-complexity detection in both clinical and field settings [17]. Cas12, Cas13, and Cas14 enzymes exhibit unique collateral cleavage activity, enabling signal amplification without the need for complex instrumentation, thereby improving detection efficiency [7]. Specific High-Sensitivity Enzymatic Reporter UnLOCKing (SHERLOCK) is a notable CRISPR-based system capable of detecting RNA or single-stranded DNA targets at very low concentrations [12]. This amplification-based platform utilises isothermal amplification and collateral cleavage of reporter molecules to generate fluorescent or lateral-flow readouts suitable for point-of-care applications [19]. SHERLOCK can detect viral genomes, antimicrobial resistance markers, and oncogenic transcripts with high analytical sensitivity, supporting early molecular surveillance in clinical practice. Optimised versions of this platform have further improved detection limits through multiplexed guide RNA design and reaction optimisation [14].

DNA Endonuclease-Targeted CRISPR Trans Reporter (DETECTR) is another CRISPR-based assay that employs Cas12-mediated collateral cleavage to rapidly identify double-stranded DNA sequences [20]. This platform facilitates the detection of viral DNA, oncogenic mutations, and host genomic alterations associated with hereditary disorders [16]. DETECTR produces quantifiable fluorescent or lateral-flow results within minutes, offering a rapid alternative to conventional PCR-based methods. Cas12-based systems demonstrate high performance in detecting viral pathogens with conserved nucleic acid targets, particularly in acute clinical settings [22]. CRISPR-based diagnostic systems have been integrated into portable and automated microfluidic platforms, enhancing clinical applicability by reducing sample preparation steps and streamlining workflows [18]. These miniaturised systems enable rapid extraction, amplification, and detection of nucleic acids within a compact analytical device, making them suitable for decentralised healthcare settings [23]. CRISPR-based assays contribute to improved outbreak surveillance, personalised therapeutic decision-making, and diagnostic testing for emerging diseases [4]. Ongoing advancements in enzyme engineering, guide RNA design, and signal detection chemistry are expected to further expand the clinical utility of CRISPR-based diagnostic technologies across diverse medical fields.

Gene therapy and genome editing as emerging therapeutic tools

Genome editing and gene therapy have transformed therapeutic strategies by enabling direct manipulation of genetic material to correct underlying molecular defects [9]. Gene therapy involves the insertion of functional genetic sequences to restore or enhance tissue function in inherited or acquired diseases [24]. Adeno-associated viruses and lentiviruses are the most commonly used viral vectors; however, non-viral delivery systems such as lipid nanoparticles are considered less immunogenic and allow for a broader dosing range [14]. Despite their widespread use, viral vectors such as lentiviruses present significant safety concerns, including risks of insertional mutagenesis, unintended genomic integration, and potential cytotoxic effects at the cellular level, which may contribute to oncogenic transformation or disrupted gene regulation (2024-2026). The choice of delivery system depends on target tissue characteristics, duration of expression, and safety considerations related to genomic integrity [9]. Genome editing technologies offer increased specificity by directly targeting endogenous DNA for modification. CRISPR-Cas systems, TALENs, and zinc finger nucleases introduce controlled strand breaks that activate cellular repair mechanisms, enabling mutation correction or silencing of pathogenic genes [15]. CRISPR-Cas9 has emerged as a leading platform due to its programmability via guide RNA and its versatility in targeting multiple genomic loci. Base editors and prime editors further enhance precision by enabling nucleotide substitutions or sequence modifications without inducing double-strand breaks, thereby reducing the risk of genomic instability [6]. However, accumulating evidence indicates that off-target activity, large unintended deletions, chromosomal rearrangements, and residual genomic instability remain critical challenges, limiting the reliability and scalability of these approaches in clinical settings (2024-2026).

Therapeutic applications of these technologies span haematological, ocular, metabolic, and oncological diseases. Edited haematopoietic stem cells have demonstrated significant potential in β-hemoglobinopathies through the reactivation of foetal haemoglobin expression. Viral vector-based gene augmentation has shown clinical success in improving functional protein expression in inherited retinal disorders. Genetically engineered immune cells expressing chimeric antigen receptors (CAR-T cells) are designed to selectively target malignant cells, improving outcomes in refractory cancers [18]. Neuromuscular and enzyme deficiency disorders can also be addressed through targeted genetic correction to restore essential protein function. Advancements in gene therapy and genome editing highlight the emergence of highly precise, molecularly targeted interventions [25]. Nevertheless, the biological complexity of gene replacement and editing necessitates a cautious approach, as long-term safety data remain limited and unintended cellular responses, immune activation, or delayed oncogenic effects cannot be fully excluded (2024-2026). Continuous longitudinal monitoring, rigorous safety validation, and ethical oversight are essential to ensure controlled clinical translation. Clinical feasibility continues to improve with ongoing developments in delivery systems, nuclease specificity, and off-target effect assessment. The expanding application of these technologies reflects their growing potential across a wide range of medical conditions [9]. Table 3 provides a concise comparison of gene therapy and genome editing in terms of mechanisms, delivery platforms, precision, and therapeutic applications.

**Table 3:** Comparative Features of Gene Therapy and Genome Editing CRISPR: Clustered Regularly Interspaced Short Palindromic Repeats, TALENs: transcription activator-like effector nucleases, ZFNs: zinc finger nucleases

Feature	Gene therapy	Genome editing	References
Mechanism	Gene addition or replacement	Targeted DNA modification	[26]
Delivery	Viral/non-viral vectors	CRISPR, TALENs, ZFNs	[22]
Outcome	Transient or durable expression	Permanent genomic change	[8]
Precision	Moderate	High	[24]
Key uses	Retinal and lysosomal storage disorders	Hemoglobinopathies, cancer cell modification	[13]

Biopharmaceuticals and monoclonal antibody therapies

Biopharmaceuticals have introduced novel therapeutic modalities through a new generation of engineered biological molecules that specifically regulate disease pathways [25]. These agents include recombinant proteins, therapeutic enzymes, cytokine modulators, fusion proteins, and nucleic acid-derived constructs, each designed to replicate or modulate endogenous physiological functions [27]. Recombinant systems enable the controlled production of structurally uniform therapeutic proteins, allowing effective intervention in metabolic disorders, immune dysfunction, and cancer. Their increased specificity and predictable pharmacokinetic profiles contribute to reduced systemic toxicity compared to conventional small-molecule drugs [5]. Monoclonal antibody therapy represents a major component of biopharmaceutical innovation. These antibodies bind to specific antigens on malignant cells, pro-inflammatory mediators, or pathogens, resulting in targeted biological effects such as receptor blockade, pathway inhibition, immune activation, or direct cytotoxicity [3]. The development of advanced antibody formats, including humanised, fully human, and bispecific antibodies, has enhanced antigen-binding affinity while reducing immunogenicity [20]. These therapies have significantly advanced disease management by enabling precise targeting of complex signalling pathways.

Further therapeutic expansion is achieved through antibody-drug conjugates, which combine cytotoxic agents with monoclonal antibodies to enable targeted delivery to antigen-expressing cells [15]. Inhibitors of programmed cell death protein-1 (PD-1), programmed death-ligand 1 (PD-L1), and cytotoxic T-lymphocyte-associated antigen 4 (CTLA-4) have transformed immuno-oncology by restoring effective T-cell-mediated immune responses against tumours. Biopharmaceutical agents that reactivate enzymes, neutralise cytokines, or block pathogen entry are also effective in treating metabolic, inflammatory, and infectious diseases [24]. Advancements in glycoengineering, formulation design, and targeted delivery systems continue to enhance the therapeutic efficacy and clinical applicability of biopharmaceuticals. These innovations enable precise modulation of molecular pathways underlying diverse human diseases [26].

Nanobiotechnology in diagnostics and targeted therapeutics

Nanobiotechnology utilises nanoscale materials engineered to enhance diagnostic precision and targeted therapeutic delivery [16]. Nanoparticles possess versatile physicochemical properties, including size, surface chemistry, and ligand-binding capacity, enabling selective interaction with specific tissues, biomarkers, or intracellular targets [27]. The evolving landscape includes metallic nanoparticles, lipid-based nanocarriers, polymeric systems, and dendrimer platforms; however, it is important to note that many of these remain in preclinical or early translational stages, with only a limited subset achieving regulatory approval. Clinically approved nanoplatforms are primarily lipid nanoparticles (e.g., mRNA vaccine delivery systems) and certain polymer-based or liposomal formulations used in oncology and infectious disease management [22]. The application of nanomaterials in diagnostics is driven by their ability to amplify signals and extend detection limits [25]. Binding of nanoparticles to target molecules generates optical, fluorescent, or magnetic responses, facilitating rapid detection of tumour markers, microbial components, and circulating metabolites [13]. Nanoparticle-based contrast agents also enhance imaging resolution in magnetic resonance imaging, computed tomography, and fluorescence-guided techniques, thereby supporting early lesion detection and efficient monitoring of therapeutic response [28]. Nanocarriers used in targeted therapeutic strategies offer advantages such as controlled drug release, improved stability, and selective tissue localisation [7]. Therapeutic agents can be transported across biological barriers using lipid nanoparticles, polymeric micelles, and mesoporous silica particles, enabling accumulation in malignant, inflamed, or infected tissues [22]. Despite these advancements, several nanoparticle systems such as dendrimers, metallic nanoparticles, and mesoporous silica carriers are still under investigation, with ongoing studies required to establish long-term safety, biocompatibility, and large-scale clinical feasibility. Targeting is further enhanced through surface functionalisation with ligands, peptides, antibodies, or aptamers that bind to overexpressed receptors, thereby reducing off-target effects and improving localisation of therapeutic action. Spatial control of drug release is achieved through stimuli-responsive nanocarriers that respond to pH, redox conditions, or enzymatic activity [29].

**Figure 2:**
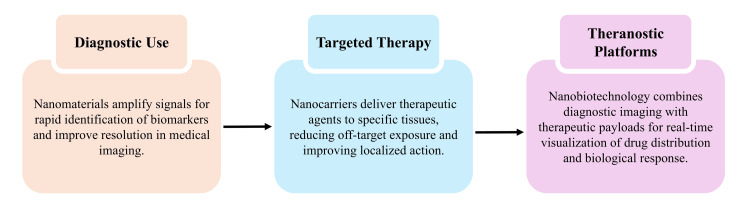
Applications of Nanobiotechnology in Modern Biomedical Practice Created by authors using Microsoft PowerPoint

Theranostic systems integrate diagnostic imaging with therapeutic delivery, enabling real-time monitoring of drug distribution and biological response. This integrated approach supports dynamic treatment planning in oncology, metabolic disorders, infectious diseases, and cardiovascular conditions [9]. Continuous advancements in nanofabrication, surface engineering, and biocompatibility assessment are expanding the clinical applicability of nanotechnology. These developments contribute to the creation of highly specific diagnostic platforms and precisely targeted therapeutic systems for complex disease conditions [21]. Figure 2 illustrates the major applications of nanobiotechnology, highlighting its roles in diagnostics, targeted therapy, and integrated theranostic platforms.

Immunotechnologies and vaccine innovations for disease control

Advancements in immunotechnologies have been driven by platforms designed to enhance pathogen recognition, optimise antigen delivery, and induce sustained and effective protective immunity [30]. Molecular engineering and innovations in delivery systems have accelerated vaccine development and improved clinical outcomes across a wide range of infectious diseases. These approaches aim to achieve controlled immune activation, reduced reactogenicity, and broad population applicability [31]. mRNA vaccines have emerged as a central component of modern immunisation programs. Messenger RNA encoding synthetic antigens is encapsulated within lipid nanoparticles, which protect the molecule and facilitate cellular uptake [16]. Once inside the cell, translation produces antigenic proteins that stimulate both humoral and cellular immune responses. The flexibility of sequence design allows rapid adaptation to genetic variations in circulating pathogens, providing a responsive platform for emerging infectious threats [32]. However, experiences from recent emergency deployment scenarios have highlighted the need to balance rapid development with rigorous clinical evaluation. Accelerated approval pathways, such as emergency use authorisations, may limit the duration of phase I-III trials, increasing the risk of rare adverse events, including vaccine-induced thrombotic thrombocytopenia observed in certain viral-vector platforms, thereby underscoring the importance of comprehensive safety assessment prior to widespread use (2024-2026).

The scope of immunisation strategies has expanded with recombinant vaccine technologies, including viral-vectored constructs, purified protein subunits, and virus-like particles [33]. Viral vectors deliver antigens using non-replicating carriers to elicit strong cellular immune responses. Protein subunit vaccines contain highly purified antigenic components, often combined with adjuvants to enhance immune response and durability [34]. Virus-like particles mimic the structural properties of pathogens without containing genetic material, resulting in high immunogenicity and favourable safety profiles [35]. Immunomodulatory adjuvants further enhance vaccine efficacy by improving antigen presentation, modulating cytokine responses, and directing T-cell differentiation [27]. Toll-like receptor agonists, nanoparticle-based adjuvants, and cytokine-mimetic systems strengthen both innate and adaptive immune responses, particularly against rapidly spreading or mutating pathogens [36]. Preclinical optimisation has therefore become increasingly critical, with advanced animal models and organ-on-a-chip systems being utilised to predict systemic inflammatory responses, immune cross-reactivity, and toxicity profiles prior to human trials (2024-2026). Ongoing advancements in next-generation vaccines are supported by progress in antigen design, delivery optimisation, and immune profiling. These innovations contribute to improved outbreak control, disease prevention, and global preparedness for emerging infectious diseases [22]. In parallel, post-market surveillance frameworks have evolved toward “pharmacovigilance 2.0,” integrating artificial intelligence and large-scale real-world data analytics to enable early detection of delayed or rare adverse effects across diverse populations (2024-2026). Furthermore, future preparedness strategies increasingly emphasise the development of standardised vaccine platform templates that undergo extensive pre-pandemic safety validation, ensuring that only antigen-specific sequences require modification during outbreaks while maintaining a well-characterised safety profile of the delivery system. Table 4 provides a comparative overview of key immunotechnology and vaccine platforms.

**Table 4:** Overview of Immunotechnology and Vaccine Platforms mRNA: messenger ribonucleic acid, HPV: human papillomavirus, TLR: Toll-like receptor

Platform	Core mechanism	Delivery design	Dominant immune profile	Main use	References
mRNA vaccines	In vivo antigen expression	Lipid nanoparticles	Strong humoral + cellular	Viral outbreaks	[37]
Viral vectors	Antigen introduction via non-replicating carriers	Adenovirus, poxvirus	Potent cellular responses	Respiratory/systemic infections	[25]
Protein subunits	Purified antigen delivery	Adjuvant-based formulations	Durable antibody responses	Chronic infections	[19]
Virus-like particles	Structural mimicry	Self-assembled particles	High immunogenicity	HPV, hepatitis	[38]
Adjuvant systems	Immune pathway enhancement	TLR agonists, nanoparticle adjuvants	Tailored cytokine activation	Broad vaccine strengthening	[32]

Regenerative medicine and stem cell-based therapeutics (concise version)

Regenerative medicine has emerged as a critical field focused on restoring damaged tissues through engineered constructs, molecular modulation, and therapeutic stem cell applications [39]. This domain addresses conditions associated with degenerative diseases, trauma, ischaemic injury, and inherited defects by promoting cell renewal, structural repair, and functional recovery [31]. These strategies are fundamentally based on the ability of stem cells to self-renew, differentiate into specialised lineages, and respond to microenvironmental signals [40]. Embryonic stem cells and induced pluripotent stem cells provide pluripotent platforms capable of generating diverse cell types, including cardiomyocytes, neural precursors, hepatocyte-like cells, and musculoskeletal lineages [29]. Induced pluripotent stem cells offer the advantage of patient-specific derivation, reducing the risk of immune rejection and enabling personalised therapeutic approaches [22]. Controlled differentiation protocols and rigorous quality assessment ensure the stability and functional integrity of derived tissues.

Adult stem cells provide additional therapeutic benefits, including lineage specificity and lower tumorigenic potential. Mesenchymal stem cells, haematopoietic stem cells, and neural stem cells contribute to tissue repair by secreting trophic factors, modulating immune responses, and homing to sites of injury [14]. Mesenchymal stem cells are particularly notable for their anti-inflammatory and regenerative properties in conditions such as osteoarthritis, myocardial infarction, chronic soft tissue injuries, and immune-mediated disorders [31]. Tissue engineering further enhances regenerative potential through the use of biomaterial scaffolds that mimic the extracellular matrix. Hydrogels, biodegradable polymers, and decellularised matrices support cell adhesion, migration, and differentiation, facilitating the formation of structurally and functionally mature tissues. Integration of vascularisation strategies and biomechanical conditioning further improves tissue viability and functional integration [41]. Gene modification approaches enhance stem cell survival, correct pathogenic variants, and improve cellular performance in adverse microenvironments. These advancements expand therapeutic possibilities for inherited and degenerative diseases [34]. Continued progress in cell engineering, scaffold design, and safety evaluation is strengthening the role of regenerative medicine as a powerful therapeutic domain with significant clinical potential [37].

AI and bioinformatics in precision medicine

Precision medicine is fundamentally supported by machine learning, deep learning, and advanced computational modelling in conjunction with bioinformatics, which enable the analysis of complex biological data to generate personalised clinical insights [35]. The increasing volume of genomic, transcriptomic, proteomic, metabolomic, imaging, and clinical data necessitates advanced computational approaches to identify molecular and phenotypic patterns associated with disease onset, progression, and therapeutic response [33]. Machine learning and deep learning technologies facilitate predictive analysis by correlating multi-omic signatures with specific clinical states [24]. These models classify disease subtypes, estimate risk scores, and predict therapeutic outcomes through high-dimensional data analysis [28]. Neural networks further enhance diagnostic accuracy in radiology and pathology by detecting subtle structural features in imaging data, enabling early identification of malignant, infectious, or degenerative conditions. Integration of molecular data with clinical parameters improves the precision and reliability of treatment planning [39].

Bioinformatics provides essential tools for sequence alignment, variant annotation, epigenomic profiling, structural prediction, and pathway analysis. These approaches identify functional mutations, regulatory elements, and disrupted molecular pathways associated with disease phenotypes [42]. Network-based analyses of genes, proteins, and metabolites enable the construction of interaction maps that support the identification of therapeutic targets and biomarker panels. Bioinformatic methods also enhance understanding of population-level genetic risk and variability in drug response [11]. Integrated machine learning- and deep learning-driven bioinformatics systems support optimised therapeutic strategies by modelling drug-drug interactions, predicting off-target effects, and facilitating drug repurposing. Predictive modelling frameworks are increasingly applied in clinical research to stratify patient cohorts, predict clinical endpoints, and optimise trial design, thereby improving efficiency and accuracy in therapeutic development pipelines [43]. The integration of these technologies into clinical workflows enhances early diagnosis, prognostic stratification, and precision-guided therapy. Continued advancements in algorithm validation, data harmonisation, and model interpretability will further strengthen the role of machine learning, deep learning, and predictive computational models in advancing precision medicine.

Discussion

The integration of biochemical and biotechnological platforms reveals significant synergistic potential, particularly through the convergence of multi-omics profiling, biosensing technologies, and machine learning-driven analytics, which collectively enhance diagnostic resolution and therapeutic precision. For instance, molecular diagnostics and bioinformatics frameworks enable high-resolution disease stratification, while nanobiotechnology and biopharmaceutical systems facilitate targeted therapeutic delivery, creating a complementary diagnostic-therapeutic continuum. Similarly, genome editing technologies combined with regenerative medicine approaches provide opportunities for both molecular correction and functional tissue restoration.

Despite these synergies, notable contradictions and challenges persist across platforms. High-sensitivity molecular diagnostics often outpace the availability of equally precise therapeutic interventions, leading to gaps between disease detection and effective treatment. Variability in biomarker expression across populations complicates standardisation, while discrepancies in scalability and cost between advanced diagnostic tools and therapeutic systems limit uniform clinical adoption. Furthermore, while technologies such as CRISPR and nanocarriers demonstrate high specificity, concerns regarding off-target effects, long-term safety, and biological variability introduce uncertainty in their widespread clinical translation.

Translational barriers remain a critical limitation. These include infrastructural constraints, high implementation costs, regulatory heterogeneity, and the requirement for specialised technical expertise. In addition, insufficient longitudinal clinical data and limited large-scale validation studies restrict confidence in long-term efficacy and safety across diverse patient populations. Integration across platforms also presents challenges in data harmonisation, interoperability, and standardisation of analytical pipelines, particularly in multi-omics and machine learning-based systems.

Future research should prioritise the development of unified regulatory frameworks, scalable and cost-effective technological solutions, and robust multi-centre validation studies. Greater emphasis is required on longitudinal safety monitoring, particularly for gene editing and nanotechnology-based interventions. Advancements in predictive modelling, data integration architectures, and interoperable bioinformatics systems will be essential to fully realise the potential of precision medicine. Additionally, interdisciplinary collaboration across molecular biology, computational science, engineering, and clinical practice will be critical for bridging the gap between experimental innovation and routine clinical implementation.

Limitations and Future Directions

This review synthesises current biochemical and biotechnological developments; however, several limitations exist. Heterogeneity in the available literature, limited large-scale validation of emerging technologies, and insufficient long-term safety data constrain a comprehensive assessment of clinical readiness. The generalisability of these innovations is further affected by accessibility challenges, cost constraints, and infrastructural disparities across healthcare systems. Additionally, the rapid pace of technological advancement may result in the exclusion of the most recent developments, thereby limiting the scope of this review.

Future research should focus on developing standardised evaluation frameworks, conducting large-scale multi-population validation studies, and implementing longitudinal clinical monitoring to improve reliability and safety assessment. Emphasis on scalable production, cost-effective implementation, and adaptation of these technologies in resource-constrained settings will enhance their global applicability. Further advancements in data harmonisation, computational analysis, and multi-platform interoperability are essential to improve diagnostic accuracy and therapeutic precision. Strengthened interdisciplinary collaboration across molecular science, engineering, and clinical domains will be critical for translating these innovations into widespread clinical practice.

## Conclusions

The combination of advanced biochemical and biotechnological methods has significantly transformed diagnostic and therapeutic approaches in modern medical practice. Molecular profiling, multi-omics platforms, biosensing systems, genome modulation technologies, biopharmaceutical agents, nanobiotechnology tools, regenerative therapies, and artificial intelligence-driven analytics collectively enhance the ability to detect diseases at earlier stages, characterise pathological complexity with greater precision, and deliver more targeted treatments. The benefits of these technologies are diverse, including improved biomarker identification, minimally invasive monitoring, highly specific molecular interventions, and the restoration of dysfunctional tissue functions. The integration of these innovations supports personalised treatment strategies aligned with detailed molecular and biological insights. The continued clinical implementation of these approaches indicates the potential for improved outcomes across oncological, infectious, metabolic, cardiovascular, neurological, and inflammatory diseases. Further advancements in safety evaluation, delivery system engineering, computational analysis, and multi-platform integration are expected to enhance the impact of these technologies on global healthcare systems. This review underscores the critical role of biochemical and biotechnological advancements in enabling precision-guided diagnostics, optimising therapeutic design, and expanding the scope of personalised medicine.
